# Book Reviews

**Published:** 1823-06

**Authors:** 


### CRITICAL ANALYSIS

OF

### ENGLISH AND FOREIGN LITERATURE

RELATIVE TO THE VARIOUS BRANCHES OF

### Medical Science.

Quæ laudanda forent, et quæ culpanda, vicissim

Illa, prius, cretâ; mox hæc, carbone, *notamus.*—PERSIUS.

### DIVISION I.

### ENGLISH.

ART. I.—*Elements of the Theory and Practice of Physic, designed for the Use of Students. Vol. I. Acute Diseases. Vol. II. Chronic Diseases.* By GEORGE GREGORY, M.D. Licentiate of the Royal College of Physicians in London, Physician to the Small-Pox and Vaccination Hospital at St. Pancras, and senior Physician to the St. George's and St. James's Dispensary.—8vo. pp. 402, and 450. Burgess and Hill, London. 1820 and 1823.

THERE are very few works of a systematic nature which fall within the range of a periodical reviewer, whose proper object appears to us to consist, not in recapitulating the first principles of our profession, but in laying before his readers all that is new or important in the publications of the day. On this account we have omitted to notice some recent works,—not from thinking them undeserving of attention, but because, from their nature and extent, they appear to us as little capable of complete analysis as the Encyclopedia Britannica or Johnson's English Dictionary. From these remarks, our readers will perceive that some motive of unusual strength must have influenced us in selecting for review a book precisely of the kind we have pointed out as unfit for the purpose. We wish, then, to introduce the "Elements of the Theory and Practice of Physic" to our readers, because we are acquainted with no publication of this nature which we can so confidently recommend to the student,—for whom it is not merely designed, but really fitted; none in which so much of practical importance is blended with so little of questionable theory. Here our praise ends; because, although well assured that we never have presumed to censure any one from the disgraceful motive of personal hostility, yet we cannot feel quite so certain that the reviewer may not sometimes be influenced by feelings of an opposite nature, and such as, in the present instance, might make high encomiums be received with suspicion, and looked upon with distrust. Our readers may possibly have remarked, that we deal but sparingly in complimentary expressions towards those authors whose works we analyze; and the reason is, that we have not the vanity to suppose our praise of consequence to any author who really deserves it, while to the reader it must be indifferent—if not fatiguing.

In criticising a work, it is always but fair to keep in view the object and design of the author; it would, therefore, be obviously unjust to censure, as an imperfect *system*, that which makes no pretensions to this imposing title. In this we think our author has shown his good sense. Indeed, in reviewing the history of medical writings, we are not so much astonished at the complete and universal failure of all systems, as at the hardihood of those who attempt this dangerous species of scientific ambition. Let all such take warning by the fate of CULLEN:—never did any one raise a more beautiful fabric;—never was any system supported by more acuteness of reasoning, or more elegance of language;—never any teacher more successful in gaining proselytes! Had his pathological views been as sound as his delineations of disease are to this hour correct and graphic, all systematic authors who have since written might have been spared the vexation of fruitless and abortive labour.

The work before us, following the arrangement of Cullen so far as it is convenient, is divided into two parts, the first of which treats of *acute*, and the second of *chronic* diseases; a division to which objections might justly be made, were it not more easy to point out defects than to suggest their remedy. Of this the author seems to have been aware, and prudently disarms criticism by admitting its imperfection. "These distinctions are not to be considered in any other light than as artificial boundaries; or as beacons which may direct the student while in the path of education, but which may be neglected when his object is attained. It will hereafter be shown that acute and chronic, local and constitutional, diseases are blended together in an infinite variety of ways, which it is in vain to attempt to unravel by the most ingenious contrivances of an artificial system." Notwithstanding this saving clause, we cannot quite reconcile ourselves to the incongruity of finding *chronic* bronchitis, *chronic* rheumatism, &c. among the *acute* diseases: neither are we persuaded that tetanus and hydrophobia are well placed among the chronic. "These, however, (we are informed,) must be viewed as exceptions to a general rule."

The *first* part is arranged as follows:—Class i. Fevers. Class ii. The Exanthemata, or Eruptive Fevers. Class iii. Phlegmasiæ, or Inflammatory Diseases. Class iv. Hemorrhagies.

The *second* part consists of—Class i. Chronic Diseases of the Encephalon. Class ii. Chronic Diseases of the Thorax. Class iii. Chronic Diseases of the Chylopoietic Viscera. Class iv.

Chronic Diseases of the Urinary and Uterine Systems. Class v. Chronic Constitutional Diseases.

As it is alike inconsistent with the limits and object of this Journal to attempt an analysis of the multifarious topics embraced in this list, we shall select one disease from each part as the subject of our remarks,—viz. Pneumonia and Dyspepsia; affording a fair illustration of what is generally understood by an acute and chronic disease.

By *pneumonia*, the author informs us, is understood the existence of acute inflammatory action in any of the structures within the thorax; while the different species of the disease described by nosologists are derived from the particular tissue affected, or supposed to be so. These are, the serous covering of the lungs,—the mucous lining of these organs, and the parts contained between these two membranes; lastly, the pericardium. After remarking, that thoracic inflammation is characterized by fever, pain in some part of the chest, difficulty of breathing, and cough, all variously modified according to the part affected, he thus describes the disease in its most acute form,—viz. *pleurisy*.

"An acute pain of the side, highly aggravated on full inspiration, is the leading characteristic of this disease. The respiration is short and hurried, and is generally performed with most difficulty when lying on the side affected. A hard and short cough is always present; and, as it aggravates the pain, it is stiffled as much as possible by the patient. At first it is commonly *dry*,—that is to say, without expectoration. The accompanying fever is urgent. The pulse is frequent, strong, and *hard*. The tongue is loaded with a thick fur. Thirst, restlessness, a hot skin, and a scanty and high-coloured state of the urine may be noticed. The concurrence of these symptoms precludes all possibility of ambiguity as to the nature of the disease, or the requisite means of relief. When blood is drawn from the arm, it will be found *cupped* and buffy."

Various authors have subdivided inflammation of the serous membrane lining the chest into different species, according as the inflamed portion happens to cover the lungs, or the ribs, or the diaphragm. These, however, Dr. Gregory omits even to mention; nor can we regard such omission as a loss: for, although it may be true that particular portions of the pleura are sometimes affected, without the disease spreading to the neighbouring parts, yet we regard any attempt to ascertain, during life, the exact seat of the inflammation as altogether useless. For example, would not any man be justly regarded as visionary, who, in any particular case, would take upon himself to say that the pleura lining the ribs was inflamed, but the corresponding portion which covers the lungs was not? But besides, however plausible such distinctions may appear in the closet, they are utterly and totally useless at the bedside of the patient; for, while a certain degree of inflammation exists, the same means must be employed, under whatever name it may be arranged in the Nosology. When, however, a different texture is affected, the symptoms undergo important modifications.

"When the mucous membrane lining the larger branches of the bronchia is affected by acute inflammation, the following is the character of the symptoms. It may be right first to mention, that this form of thoracic inflammation is less frequent than the preceding, though on the whole more dangerous. The most urgent symptom is a sense of tightness or constriction about the chest, often referred very unequivocally to the precise seat of the disease. Respiration is hurried, and accompanied by a wheezing in the throat; but it can often be performed without increasing the uneasiness of the patient. There is cough, which from the first is attended with some degree of expectoration. The general febrile symptoms are very severe. The pulse is frequent, but it often wants that fulness and hardness which characterize pleurisy. Not unfrequently it is intermitting. There is always observable a remarkable expression of *anxiety* in the countenance, generally with paleness. The functions of the brain are here more disturbed than in the common cases of thoracic inflammation. In the progress of this disease, authors have noticed that occasionally, at a particular period, the constitutional symptoms are suddenly converted from those of high inflammatory action into such as indicate extreme debility, or exhaustion.

"The substance of the lungs is often the primary seat of acute inflammation, and the term *peripneumony* is usually applied to this form of thoracic inflammation. In most of these cases, the inflammation occupies the smaller ramifications of the mucous membrane, but the proper cellular texture of the lungs will, of course, partake of the disease. The parenchyma of the lungs may also be secondarily affected. In a few cases of pleurisy, the inflammation is altogether confined to that membrane, but more commonly it implicates the neighbouring portions of the substance of the lungs. The usual symptoms of peripneumony are, an obtuse pain, sometimes referred to the side, but more usually to the sternum or epigastrium, and occasionally to the back or shoulder; impeded breathing, which is often particularly difficult in the recumbent posture; a moist cough; and fever, the character of which, however, is subject to great variety. Sometimes there is so little constitutional disturbance, so little febrile oppression, that the disease makes rapid advances before its nature is suspected. Sometimes the pulse is hard, but much more commonly it is distinguished by its fulness and *softness*. Peripneumony is often attended by a puffiness of the features, lividity about the lips and under the eyes, and occasionally head-ache; symptoms obviously referrible to the difficulty experienced in the transmission of blood through the lungs."

The prognosis in pneumonia is said to be "not unfavourable," and consequently resolution is the most frequent termination. Here we do not think that Dr. Gregory has pointed out, with sufficient precision, the modifications which our prognosis must undergo, according to the seat of the disease: for, although he informs us that, when the mucous membrane is affected with acute inflammation, it is "on the whole more dangerous," we must object to this expression, as it does not, in our judgment, convey an adequate idea of the much greater degree of danger attending acute inflammation of the mucous membrane of the pleura. The various terminations in *expectoration*, *effusion*, *empyema*, *vomicæ*, *adhesions*, and *hydrothorax*, are clearly described, as well as the more common exciting causes.

The principles of the cure are sufficiently obvious; but their application in each individual case requires a degree of discrimination which experience alone can communicate.

"1. In blood-letting, we possess a power of controling pneumonic inflammation, the efficacy of which has been acknowledged in all ages, and is obvious, indeed, to the most superficial observer: but much depends on the manner in which it is performed, the quantity of blood drawn, and the frequency of its repetition. Physicians have been struck, at all times, with the effect produced by taking the blood from a large orifice, in this and other urgent cases of local inflammation; and it certainly cannot be too strongly urged as an indispensable point in practice. The orifice should be such as to allow a pound of blood to flow in five, or at furthest in six, minutes. The quantity to be taken at one time cannot be defined with any degree of accuracy. A pound of blood may be looked upon as an average for an adult. As a general rule, it may be stated that some effect ought to be produced on the system, before the orifice is closed: either faintishness, or sickness, or diminution of pain, or of the strength of arterial contraction.

"2. In all cases of pneumonia of the least severity, bleeding from the system must be repeated; and the principal circumstances by which the frequency of its repetition is to be regulated, are the state of the symptoms, and the appearance of the blood drawn. Blood-letting is better borne in pleurisy than where the mucous membrane of the bronchia is the chief seat of disease; and, as expectoration of mucus is one of the means by which all inflammation within the chest is relieved, venesection, on several accounts, must be practised with great caution when that symptom occurs. When suppuration has commenced, copious bleedings are inadmissible; but small bleedings may then often be resorted to, with the happiest effect. Although the presence or absence of buff is not to decide our practice as to future bleeding, still, when present, it may often materially assist us in our judgment. If the blood, besides being buffy, is cupped and *fringed* at the edges, we need have little hesitation in repeating the evacuation. Should the blood appear with a flat surface of buff, and the coagulum be loose, further bleeding may indeed be still necessary, but it must be practised with more caution. In the pneumonia of infants, and occasionally with adults also, leeches and cupping may be substituted for bleeding at the arm; but the circumstances warranting this are very few.

"3. Moderate purging, by the neutral salts, is a useful auxiliary in the treatment of pneumonia; but the advantages of purging are, upon the whole, much less obvious in thoracic diseases, than in those of the head or abdominal cavity. An attempt to overcome decided thoracic inflammation by severe purging will always prove ineffectual, and often prejudicial. Refrigerant medicines, as nitre, may be employed with great propriety. A free expectoration being, as we have said, the means which nature most commonly adopts for carrying off inflammation within the chest, it might be supposed that expectorant medicines would prove useful; but the reliance to be placed upon them is very small. Antimony and squill are the only ones of this class which can be recommended. Opium is quite inadmissible during the active stages of pneumonic inflammation. Even in the more advanced periods of the disease, it must be given with extreme caution, on account of its tendency to check expectoration.

"4. Blisters are unquestionably of the greatest importance in the treatment of pneumonia; but they should not be applied while the pulse is hard, and the blood appears cupped. It is not until the tone of the system has been sufficiently lowered by venesection, that their good effects will become apparent.

"5. If the inflammation has terminated in suppuration, besides the small bleedings already recommended when the difficulty of breathing becomes particularly urgent, advantage will be derived from the continued exhibition of the tincture of digitalis. The strength of the patient must be supported by a light, nutritious diet; but wine is to be avoided. The operation of *paracentesis thoracis* is probably advisable in certain cases, both of vomica and empyema: but the observations of authors on this piece of practice are very scanty, and my experience does not enable me to supply the deficiency."

These extracts will serve as a specimen of the manner in which the author has executed his task with regard to acute diseases: we now turn to those of a chronic nature. Here we would pause for a moment, to remark on the apathy too often displayed by the branch of the profession to whom the "Elements" are addressed, with regard to this class of diseases. We see surgical pupils crowding every day to witness the performance of any capital operation, which not one in ten will ever have an opportunity of practising: indeed, the eagerness to gain admission is generally in proportion to the rarity,—that is, (so far as concerns them,) to the uselessness of the operation in question. Now all this is natural, and even commendable within proper bounds; but we fear that the zeal for surgery too often ends in the passion for what is new or uncommon, while the more really important cases of every-day occurrence are passed unheeded; and many a student, who could tie a great vessel with much dexterity, would be puzzled to heal an old ulcer. So it is in physic:—young men are too apt to regard as uninteresting those chronic cases which are marked by no prominent features, and over which remedies exert no immediate or striking effects. Yet the treatment of such diseases constitutes the true test of skill, and affords the daily bread of the successful practitioner. To all the junior members of the profession we earnestly enjoin an early and minute attention to the pathology and treatment of this class of complaints: the importance and difficulty of the investigation afford a proper field for the exertions of men ambitious of the honours of science; while their frequency fits them for those who look to the emoluments of the trade.

We return to Dr. Gregory; and his account of the best illustration we know of the preceding remarks,—viz. the history of *dyspepsia*, or indigestion. This ailment, the author observes, "is met with in every country,—in every class of society,—in every season of the year." The meaning of dyspepsia, according to its restricted signification, is limited to derangement in the functions of the stomach, without any other disease. In the work before us, the inconveniences of this limitation are pointed out, and the term is extended to those cases of indigestion which are unattended with well-marked general fever, local inflammation of the stomach, or obvious *cognizable* disease of a remote organ. Dyspepsia is further reduced to *primary* and *secondary;* the symptoms common to both being described as follows:

"The symptoms of dyspepsia are extremely diversified. They may be divided into such as are referrible to the stomach itself, or to its sympathies with other parts of the body. Among the first may be enumerated loss of appetite, nausea, pain in the epigastrium or hypochondria, heartburn; a sense of fulness, distention, or weight in the stomach; a feeling as if a ball was lodged in the œsophagus; acid or fœtid eructations; pyrosis, or the vomiting of a clear liquor, often in vast quantity; and, lastly, a sensation of *sinking*, or fluttering at the pit of the stomach. To the second head of dyspeptic symptoms may be referred, among many others, costiveness, or an irregular state of the bowels, with a morbid appearance of the evacuations; pain of the back, and turbid urine; a disagreeable taste in the mouth, especially on first waking; tooth-ache; palpitation, pulsation in the epigastrium, irregularity of the pulse; a short dry cough, and occasional difficulty of breathing; giddiness and head-ache, sometimes referred to the fore, but more commonly to the back part of the head; languor, lassitude, and great depression of spirits, with fear of death, or of impending evil.

"The tongue is very generally referred to as affording evidence of the state of the stomach; but it will often be found that the tongue is perfectly clean when the stomach is most incontestably disordered. It would seem, indeed, as if the morbid appearances of the tongue,—its fur, dryness, preternatural redness and smoothness, and its chopped aspect,—are referrible to the state of the constitution, rather than to any particular derangement in the stomach. When, however, we observe the tongue *furred and moist,* (its true character in common dyspepsia,)—that is to say, when the secretions of the mouth are depraved, we may reasonably presume that there exists a similarly disordered state of the *secretions* of the stomach."

In order to elucidate the *ratio symptomatum,* the author gives a brief outline of the physiology of the stomach; according to which, it would appear that the food requires to be mixed with certain fluids,—to be exposed to the influence of certain nerves,—to acquire an appropriate fluidity, and to be propelled with a due degree of energy. From this it is supposed that indigestion may arise from disease of the glands subservient to the stomach, (rendering the fluids vitiated or deficient, or in excess;) or from a morbid state of the nerves, either local or general; or the fault may consist in the *muscular* coat being so affected as to hurry the food too rapidly, or detain it too long; or, lastly, the fault may lie in the duodenum.

It has generally been the custom to subdivide dyspepsia according to the symptoms; a method condemned by our author, as affording no indications for the permanent removal of the complaint. To remedy this disadvantage, Dr. Gregory proposes to class the different forms of dyspepsia according to the causes which excite it. There are undoubtedly, he says, persons who appear to possess, and perhaps even to inherit, constitutional weakness of the stomach; but such cases are at least *very uncommon,* and may, without impropriety, be discarded from our present consideration." Now, whether our author has done well in omitting this form of the disease in his division, we know not; but we must protest against the accuracy of the position. We have seen too many instances of parents subject to dyspepsia having children afflicted with bad digestion, to have any doubt of the ailment being *very frequently* transmitted by hereditary predisposition. The following is Dr. G.'s classification; to which are added remarks on each variety, which our limits prevent us from being able to extract.

"*Tabular View of the Varieties of primary Dyspepsia.*

" 1. Dyspepsia from occasional overloading of the stomach.
" 2. ———— from habitual over-feeding.
" 3. ———— from habitual indulgence in spirituous liquors.
" 4. ———— from want of air and exercise.
" 5. ———— from excessive or long-continued evacuations.
" 6. ———— from anxiety of mind.

"*Tabular View of the Varieties of secondary Dyspepsia.*

" 1. Dyspepsia, symptomatic of general feverishness.
" 2. ———— ———— of habitual constipation.
" 3. ———— ———— of chronic disease of the liver.

"4. Dyspepsia, symptomatic of chronic disease of the spleen.
"5. ———— ———— of functional disturbance of the uterus.
"6. ———— ———— of obscure disease of the kidney.
"7. ———— ———— of chronic affections of the bronchia.
"8. ———— ———— of chronic cutaneous diseases."

With regard to the prognosis, Dr. Gregory states that, *in all forms of dyspepsia, it is favourable* :—we regret to say, that in our practice we have not found it so. If, by prognosis, the author means the result of the disease with respect to the life or death of his patient, then doubtless his assertion will be correct in the majority of cases; but if he alludes to the *cure* of the disease, we would ask on what principle he ventures to speak so favourably in the third variety (for example,) either of his first or second table? The truth appears to us, that much may be done in alleviating the complaint, but very little in *curing* it: withholding from a dyspeptic patient substances which disagree with him, may, and generally does, render him more comfortable, but will by no means enable his stomach to digest like that of a healthy individual. The following observations connected with this part of the subject are worthy of all attention:

"It is unnecessary to say that there is no one drug which will fulfil the great object of treatment, that of giving *tone* to the weakened stomach of a dyspeptic patient. This can be obtained only by measures calculated to avert the cause which may have excited the disease. The tone of the stomach never fails without some assignable reason, which strict inquiry will detect, and the knowledge of which will point out the proper means of relief. Nor is it often that these will fail of success, provided the patient have sufficient firmness to submit to them, and afterwards remain sensible that his health is in his own hands. The assistance of the physician, however, is very often required where the patient either cannot or will not submit to the measures which prudence dictates. In such circumstances, we must endeavour to aid the digestive process by medicines; but I would wish to impress upon the student the impropriety of trusting to them in dyspeptic cases. He should remember that almost any drug will injure digestion in a healthy state, and he should learn, therefore, to be sparing of medicine when the stomach is weakened by disease.

"In every form of dyspepsia, attention to diet is indispensable, and the patient must have regard, not to its quality only, but to its quantity. In a weakened state of the stomach, it must have little given it to do. The body is strengthened, not in proportion to the quantity of food taken in, but to that which is thoroughly digested. Differences in the habits of life will, of course, lead to important differences in the kind and quantity of diet which should be permitted to a dyspeptic patient; but the following may be regarded as rules of very general application: —It should consist in a due mixture of animal and vegetable food, but the former should be eaten only once a-day. It should be thoroughly masticated. Great varieties of food at any one time should be prohibited, as leading to an indulgence of the appetite beyond the wants of the system. Articles of difficult digestion should be carefully avoided; such as all kinds of smoked, hard, dried, salted, and long-kept meat; all those dishes where too much nutritious matter is collected in a small space,—eggs, for instance, potted meats, strong soups, and preparations of suet, fat, and butter; lastly, all raw vegetables whatever, with the exception of ripe fruits. Regularity in the hours of meals should be rigorously enjoined, and the patient directed to abstain from food at all other times."

The only part of these general remarks in which we do not entirely concur, is that which relates to animal food, which we are informed should only be eaten *once* a-day. There are, however, many cases in which there is so great a tendency in the stomach to generate acidity, that animal food alone is capable of resisting this morbid degeneration; and we are convinced, from personal experience, that it may occasionally be substituted, with much relief, for the more generally useful forms of vegetable diet.

The particular directions for individual cases being too extended for quotation, we are constrained to content ourselves with an analysis of them. In the *acute* dyspepsia, emetics are recommended; but their frequent employment is very properly condemned, as tending to weaken the stomach. In cases not of long standing, brisk purgatives are enjoined; and in habitual dyspepsia, small doses of laxatives, "just sufficient to keep up a gentle peristaltic motion through the whole alimentary canal." Bitters and similar remedies, the author is of opinion, have been too much employed, and with too little discrimination, being inadmissible in all cases attended with a *feverish* and *irritable* habit of body. In that form of the disease, however, which arises from protracted lactation, the most powerful tonics must be had recourse to,—as iron, cinchona, aromatic confection, and ammonia. Absorbents are capable of affording relief, but this is asserted to be *always transitory*. Of mercurials the author speaks as follows: "Mercurial preparations are frequently resorted to in simple dyspepsia, not as purgatives, but in small doses for their specific, or, as it is said, alterative, effect upon the secretions of the body. Three grains of the blue-pill, given at bed-time, certainly prove serviceable in many obstinate cases; but it is difficult to define under what circumstances such a plan of treatment is essentially required, or on what well-ascertained principle it operates."

Besides these remedies, there are others of obscure or doubtful agency, which are classed together under the head of *nervines*, such as oxyd of bismuth and sulphate of zinc, given in minute doses. Among the medicines which exert a decided, but hitherto ill-defined, influence upon the stomach, we would place the *hydrocyanic acid*, of which the author has made no mention, although the testimonies of Drs. Elliotson and Prout are such as to entitle it to notice, if not to commendation. We are satisfied that, in dyspeptic cases, accompanied by that form of pain to which the name of gastrodynia has been applied, the prussic acid is a remedy of considerable efficacy. Indeed, we have observed throughout that the author is somewhat too diffident in introducing any new medicine to his readers; a caution which, though in itself judicious, may be carried too far. Thus, in treating of *neuralgia*, no reference is made to the plan of treatment so successfully employed by Mr. Hutchinson, of Southwell; and, although iron is cursorily mentioned along with bark and arsenic, yet the attention is not particularly directed towards it,—nor is the *subcarbonate* even mentioned.

We have now brought to a close all the observations on Dr. Gregory's "Elements" which our limits will permit us to make. The freedom of our remarks will prove the strictness of our scrutiny; while the merits of the work will not, we trust, be judged by the scanty measure of the praise we have bestowed upon it.

---

Art. II.—*An Essay on the Medicinal Efficacy and Employment of the Bath Waters; illustrated by Remarks on the Physiology and Pathology of the Animal Frame, with Reference to the Treatment of Gout, Rheumatism, Palsy, and Eruptive Diseases.* By Edward Barlow, m.d. &c. &c.—8vo. pp. 200. Longman and Co. London, 1822.

Those who only read the title-pages of books (of which the number, we fear, is not small,) will form a very erroneous opinion of the work of which we are now about to give a sketch. It does not contain one word relative to the analysis of these celebrated waters; there is no calculation of their solid or gaseous contents; scarcely any remark even upon their temperature; and not a word relative to their previous history. To compensate, however, for these deficiencies, (if they may be so called,) this little volume contains much sound pathology, a great portion of good sense, and the whole is further recommended by the modesty and perspicuity with which it is presented to our notice. Our author's design in writing this work is explained in a short preface, from which we gather that his object is to recall towards the Bath waters that attention to which he conceives them entitled, but which has, of late years, been sensibly weakened or withdrawn: to effect this, he enters into the consideration of the pathology of those diseases in which they are beneficial. "Faithfully believing the principles advanced to be founded in truth, the author is happy in thus submitting them to the judgment of his professional brethren, from whom he has no doubt of experiencing candour and impartiality." (Pref. p. 1.) He afterwards endeavours to obviate any objections that may be urged against his arrangement and division of plethora; and he adds, "If the division adopted ensures but perspicuity, that first requisite of didactic composition, the author's object is attained. (Pr. p. 2.) The next paragraph, we conceive, demands some notice; it is as follows:

"It was chiefly in compliance with the wishes of friends, on whose judgment the author relies, that cases are given. Being little in the habit of depending on reported cases for regulating his own practice, and having ever found well-established principles the only sure guides, he would willingly have referred practitioners to their own daily experience for illustrations of the doctrines advanced; for, if not supplied abundantly from such a source, the doctrines themselves could be little worth, and scarcely suited for general adoption."

We will not here repeat the trite and hackneyed observation relative to "false facts," which no doubt has been, and very frequently still continues to be, a just ground of reproach to our profession; notwithstanding which, we must beg to observe, that doctrines and facts are equally necessary towards the perfection of our science, as throwing a mutual light upon each other: and if cases are to go for nothing, and *principles* are the only sure guides, one-half of our work is written to no purpose; a deduction which the profession in general, we are convinced, will not agree to, since we fear that, with all our self-love and vanity to boot, we never can persuade others, and scarcely even ourselves, that our labours are at all equal in merit to those of our valuable and esteemed correspondents: nay, our author himself adds numerous cases in illustration of his doctrines, which, we presume, he publishes in the belief that they will, at least in some degree, influence the practice of those who read his work; or with what design, and to what end, are they inserted at all?

Our author concludes his preface by observing, that, although some great names are directly chargeable with misrepresenting the Bath waters, he has passed them unnoticed; being content to rest their character as a remedy on the direct testimony of fact and experience.

After some preliminary remarks, our author commences with an exposition of his principles of general pathology, upon which his recommendation of the use of the Bath waters is founded; and the soundness of which, he says, a tolerably extensive practice, both in acute and chronic diseases, has confirmed. We shall quote the first paragraph, in order that we may better understand, and be enabled more easily to condense, the remaining sections of the work:

"The first main fact in animal physiology, of which every ordinary observer can take cognizance, is that the animal frame, from the period of birth to that of dissolution, is in a constant state of decay and renovation, or rather of waste and repair. The constant and unremitting exercise of those functions, the aggregate of which constitutes all that we know or can conceive of life, is attended with a physical expenditure, which our daily nutrition supplies. The nutritive matter taken into the stomach, which forms the chief support of animal life, undergoes several successive changes in its passage through the body,—is digested, assimilated to the animal juices, deposited in the several structures for their appropriate nutrition; and, finally, when no longer fitted for supporting their healthy actions, is taken up by the absorbents, and carried out of the system by the appropriate excretories.

"The various excretions are liable to considerable variations; so also is the quantity of food which any individual consumes; and still more the assimilating processes by which that which is taken into the stomach is animalised and fitted for repairing the waste of the system. If more food be assimilated than the ordinary waste of the body requires for its repair, a state of repletion must be the natural and inevitable result. But repletion may also take place under a moderate, and even abstemious, supply of food, when, from a sedentary life, inactive habits, or any defect in the functions of the several emunctories, the necessary excretions are inadequately performed."

From this it appears that his doctrines are founded in the belief of the existence of three peculiar stages of repletion, or plethora,—the first being absolutely a state of repletion from excess of nourishment, in a healthy constitution; the second also arising from repletion, but in a habit less vigorous, so that the self-adjusting powers are not so successful in effecting early restoration, and inflammatory actions are more tardy in their accession. This interval, affording symptoms of an equivocal character, and often liable to be misunderstood, he thinks has been imperfectly investigated, and not often brought under the notice of the physician sufficiently early. The last case of repletion arises from moderate nutrition and defective excretion, in which the system is not oppressed by the quantity of food, or by the first part of the process of digestion, but by the accumulation of the excrementitious matter.

We come now to a consideration of the first of these conditions, which include pure "inflammations." It is customary, he continues, to refer these diseases to an exciting cause; but here he justly remarks that, whilst, from exposure to cold, one person shall have catarrh, another rheumatism, a third inflammation of the lungs, whilst the majority will escape altogether, this effect is only produced because the body exposed is already prone to be affected, in consequence of its own previous derangement. He tells us that he has endeavoured to distinguish this particular state of an apparently healthy body, in an essay published in a cotemporary Journal,* but which we shall not here notice, because we conceive that these marks are, after all, not very clear; and we are quite sure that the bulk of mankind never will consent to be physicked for fear they should be unwell, although they do not happen to feel so. For this class of diseases, Dr. Barlow, of course, does not advocate the use of the Bath waters; and therefore we pass on to the second stage, in which the deviations from health take place gradually, and are indeed often for a long time unnoticed, irregular circulation is marked by cold feet and a variable countenance; the bowels are irregular, the appetite capricious, and both flesh and strength decline. In the earlier periods the pulse is weak, and often irregular. Sooner or later a febrile state ensues, with heat of skin and furred tongue; and often, in the course of time, some local complaint supervenes, and absorbs the attention entirely. As the weakness and irregularity of the pulse in the first stage of the disease must be distinguished from pure debility, the examination should be very accurately made; and this can only be done by a *firm pressure* on the artery, which it will often be found to resist, and, on gradually withdrawing it, to rebound against the finger with considerable force. The irregular action of the artery with regard to strength, says our author, indicates a still nearer approach to the period of inflammation, than an irregularity of frequency only. These stages may be accelerated by medical treatment.

The last stage of plethora, that arising from defective excretion, is next considered; and in this the deviation from health takes place still more gradually. This is the disease of the sedentary and the indolent. Sallowness of aspect, a dry harsh state of the skin, a low and compressible pulse, a capricious appetite, and foul alvine evacuations, with high-coloured and fetid urine, or even perspiration, mark this condition of disease; but the obstinate foulness of the bowels is the most constant symptom, and which purgatives do not correct: if, however, the constitution is so far relieved by them as to enable it to make an effort towards the febrile and inflammatory stage, small bleedings, if employed with discretion, will do more to correct the condition of the bowels than any use of purgatives; but this must not be resorted to without considerable caution, nor at too early a period, least irremediable exhaustion should result.

"I shall now (says Dr. Barlow,) resume the consideration of each

---

* *Edinburgh Medical and Surgical Journal.*

condition separately, and direct the practical treatment applicable, together with the modifications required by the supervention of those specific diseases to which they are severally liable; and under each head shall point out how far the use of the Bath waters may be rendered conducive to that re-establishment of health and strength which is the end of all medical treatment."

We come now to the medical treatment of the first condition of plethora. This our author divides into three stages: the first being the period preceding febrile excitement; the second, wherein that is unaccompanied by any local affection; and the third, where that is superadded. He admits that a knowledge of the first of these stages is chiefly necessary for the medical man; for the precautions are scarcely available for the security of mankind in general, as we have already remarked; and we cannot help considering that our author carries this particular part of his subject a little too far, inasmuch we think a reduced diet alone, or perhaps a little opening medicine, which most unprofessional people are in the habit of employing under the feelings excited by this condition, are quite sufficient to restore the balance of the constitution, without recurring to the use of the lancet. Having given this as our opinion, we shall pass on to the following passage, the truth of which we willingly accede to, and which, we firmly believe, is not sufficiently attended to in private practice.

"I may here, however, cursorily remark, that when morbid actions of an inflammatory kind have commenced, requiring direct depletion by blood-letting, it is the last portions of blood taken that afford the effectual relief; for, under high and active inflammation, twenty, thirty, forty ounces of blood, or more, may be taken without making any impression on disease, when the loss of a few additional ounces will, at length, by inducing a disposition to syncope, completely arrest the inflammatory action, and subdue effectually the violence of disease.

"In such case, if the depletion be regulated by regard to the quantity of blood taken, rather than the effects resulting; and if, under the apprehension of a larger depletion, it stop short of that relief which ought alone to set limits to it, the morbid action is left unsubdued, and much greater loss of blood will eventually be required to allay it, with the great risk of failing altogether in accomplishing this end: for it should ever be borne in mind, that in proportion to the time which morbid action of this kind has been suffered to continue unrestrained, will be the final difficulty of arresting it."

The second stage, or that of febrile action, is next treated of; but we may venture to pass this over, as the precepts, though judicious, are not novel, and the plan of treatment laid down is pretty universally understood. In advocating blood-letting, however, in certain states of active inflammation, we fear that our author has gone rather farther than, perhaps, is prudent; and yet no one has employed blood-letting with greater freedom and confidence than ourselves: still there is a most marked difference in the application of this practice in different classes of life, in different conditions of society, and even of sex; and, unless this be taken into the account, bleeding, efficacious and powerful as it is, may yet be carried to a hurtful extent. We may perhaps be accused of timidity; but, as our remarks are chiefly addressed to the young practitioner, we are disposed to urge a line of conduct savouring of prudence and caution, rather than to add a spur to the adoption of measures which our juniors are generally well disposed to pursue, without drawing those nice distinctions which the experienced physician often observes, without being actually able to explain by mere words. There is, however, much truth in what Dr. Barlow says respecting the different conditions of those who live in London and in the country; and the whole passage is full of good sense, though urged, as we have before said, a little too strongly.

In considering the medical treatment of the second condition of plethora, our author observes, that the apparent state of feebleness which accompanies the commencement of this condition is followed usually by increased action, and some local malady becomes superadded. A hard frequent pulse, hot skin, and a white tongue, mark the formation of the second stage, and which often last for months, or even years, without any specific disease ensuing. This condition Dr. Barlow calls "constitutional inflammation," and he advocates blood-letting: and so partial is he to this remedy, that he is almost inclined to recommend the old-fashioned practice of bleeding at spring and fall, as preventive of disease. (p. 49.)

Under this second condition of plethora, he conceives that the congestions or other derangements of minute structure cannot be remedied without some effort of the constitution; and he therefore founds his practice upon this view of the subject, prescribing small bleedings and moderate excitement, watching the time when increased action in the pulse will justify direct depletion. The first bleedings he recommends under these circumstances are small, from four to six ounces; and he gives two or three instances where quantities of blood, to the amount of 209 and 106 ounces, were severally taken from apparently weakly and delicate subjects, at twelve and seven successive evacuations, the strength improving in each instance under this practice. The early symptoms in each were those of seeming debility.

In judging of the approach of the inflammatory or febrile stage, our author is chiefly influenced by the state of the tongue, which presents a peculiar whiteness, "seemingly belonging to the substance of the tongue itself," and which he has seen to disappear immediately after a full bleeding. (p. 53.)

In those cases where excitement is in the first instance required to accelerate the second or febrile stage, so its continuance is called for to support the constitution, as well as when these efforts are on the decline; and here we find the first recommendation of the Bath waters for that purpose. It thus appears that blood-letting is the remedy which our author chiefly relies on in these different conditions of the system; but next in importance to this he places purging. The medicines of this class he distinguishes into three kinds: those simply evacuating the feculent contents of the bowels; those which lessen arterial action, by abstracting fluids from the body; and those which require to be employed when the mucous secretions are so morbid as to excite or aggravate disease, or when they are too copiously generated in consequence of increased actions in the vascular system. The application of these remarks he leaves to the practitioner; and we find nothing novel in the practical remarks he has made upon this subject. He urges a careful inspection of the fæces, which, in these cases of chronic disease, we believe to be very important, and not enough attended to.

The next head, or division, treats of Gout, which, says our author, admits of signal relief by the judicious use of the Bath waters, notwithstanding what some eminent writers have alleged. We need not here repeat the well-known symptoms of this intractable disease, upon which so much has been written with so much confidence, and to so little purpose. Dr. Barlow thinks that the "complicated histories of gout" are only modifications of the more simple series of phenomena described in the foregoing part of his work: "they exhibit essentially only a plethoric state of constitution, passing into one of febrile excitement, and ending in specific partial inflammation." (p. 71.) In conformity with this opinion, our author, of course, condemns the use of specific remedies, and founds his treatment upon its connexion with the three conditions of plethora already noticed. After some remarks upon the hereditary disposition to gout, he continues thus—"When gout arises, for the first time, in a constitution otherwise healthy and vigorous, consequently under what I have described as the first condition of plethora, there can be no reason for not employing the same constitutional treatment that would be resorted to under any other active inflammation." He therefore bleeds largely in the first instance; purges with calomel and antimony, followed by a saline cathartic; and uses the other general remedies employed in the treatment of inflammatory diseases, repeating the venesection according to the circumstances of the case,—the only distinction he makes between this attack and any of the simple phlegmasiæ being in the topical treatment: for in gout he does not think it right to abstract blood from the inflamed part, nor to employ repellents; the practice which has been recommended at various times, and revived of late years, of subduing the local complaint, without regarding the constitutional derangement, being, in his opinion, very hazardous, and at variance with sound pathology.

Having subdued the paroxysm, the next point is to attend to the premonitory symptoms which mark the renewal of plethora; and this, in gouty habits, is rendered comparatively easy by the deranged condition of the stomach, which so usually precedes the attack. This, if watched, and treated by judicious depletion and a regulated diet, will anticipate the threatened attack; and he doubts not of the perfect practicability of finally extinguishing the disposition to gouty action.

In discussing the merits of the colchicum, we find the following remarks:—Blood-letting, says Dr. Barlow, should, when inflammation is high, always precede the exhibition of this remedy; but not when arterial action is more moderate, and direct depletion therefore becomes questionable. The tincture of the seeds is the form of preparation which our author prefers, as he conceives it to be more uniform in its strength, and more certain in its operation. With regard to the mode of exhibition, it may be either given in full doses, so as to purge actively, or in divided doses frequently repeated: for the former purpose, "a dram and a half or two drams" of the tincture of the seeds, administered at night, and repeated if necessary in the morning, will generally purge briskly; its use may then be continued in doses of twenty minims three times a-day, in any saline medicine.

The preceding observations appear to apply to gout attacking vigorous habits, and where arterial action runs high; for our author is careful to inculcate that every case of gout will not bear this active mode of treatment: neither does this class of gouty patients require the aid of the Bath water, as there is no necessity for their assistance in accelerating the paroxysm. Neither is the convalescence sufficiently tedious to render a journey to Bath necessary.

The various modifications of gout, arising from its connexion with the second and third condition of plethora, next occupy our attention; and, as these conditions pass insensibly into each other, and co-exist in every conceivable proportion, "I shall consider (says Dr. Barlow,) the remaining varieties of gout, not as distinguished into classes, but as forming a descending series from that in which considerable nutritive plethora exists, with little excrementitious repletion, to that where the latter condition so predominates as to render any intermixture of nutritive plethora scarcely discernible. (p. 88.)

This leads to the *medical treatment of the third condition of plethora.* The symptoms indicating this condition have been already mentioned. The foul and unhealthy state of the fecal discharge is the most marked symptom, combined with wasting of the flesh and strength. Our author is aware that the symptoms constituting this state are often regarded as evidences of diseased liver, and treated accordingly; and he does not deny that they are to be met with in certain conditions of that viscus; but they are also found to be present when the liver is in no way affected, at least primarily, but merely from its being called upon to act inordinately on a vitiated mass, which nature is sedulous to purify. (p. 91.) This endeavour excites a febrile condition of the system, in the relief of which the cure of the vitiated secretion consists; and therefore, to promote the intestinal discharge by suitable purgatives, at the same time supporting the strength with a light nutritive diet, is the first indication. In proportion as the constitution rallies, the depleting system must be more active, and the diet less stimulating; and when the excitement is considered as sufficient to justify bleeding, our author considers the case in the most favourable train. In the former stage, we are told that the plan of treatment laid down by Mr. Abernethy is applicable; but to this plan Dr. Barlow thinks no auxiliary remedy better suited than the Bath waters, used both externally and internally; the former restoring the functions of the skin: and it is suggested that a vapour-bath might be still more efficacious for this purpose. The Bath waters, internally exhibited, derive their superiority, says our author, from the peculiar tonic and stimulating properties which they possess; they also promote diuresis; and, by exciting the whole vascular system, they powerfully assist the efforts of nature in forming the stage of mild febrile excitement which forwards the work of purification. The peculiar view which Dr. Barlow has taken of his subject is well explained in the following passage, which concludes this section of his work.

"From the descriptions given of the several conditions of plethora, the peculiar phenomena of each may be readily distinguished in those complex cases where the second and third are so constantly and so variously combined. Accordingly as the third condition predominates, will purging, nutritive diet, and moderate stimulation, be requisite. When the second prevails, direct depletion by blood-letting is necessary, and a more cooling regimen should be enjoined; but not the full antiphlogistic discipline so indispensable in cases of high inflammatory action.

"The combination of stimulant remedies with depletion, is a part of medical practice that seems never to have been properly discussed, though frequently noticed incidentally by practical writers, and often conspicuous in the popular and empirical treatment of diseases. It is assuredly one of the utmost importance, and will, I trust, receive some illustrations from the present work. I am the more anxious to bring this matter under consideration, because a misapprehension respecting it seems, of late years, to have had considerable influence in causing the Bath waters to be withheld from patients manifesting any slight febrile symptoms, who might nevertheless have used them with the utmost advantage. This error appears to have arisen from trusting too much to speculative reasoning, without sufficiently regarding the evidence in favour of the salutary administration of these waters, which experience had so copiously supplied."

Having discussed this point, Dr. Barlow resumes the consideration of gout, as affecting constitutions less vigorous than those previously treated of, and under some of its more complex modifications. We may pass over this division rapidly, merely observing that our author displays his usual good sense, and is anxious to show that his love for bleeding does not induce him to recommend it without considerable caution and limitation in such cases. Generally speaking, he considers local applications as hazardous; and he remarks, as a corroborating evidence of the correctness of the general principles he has advocated, that, whenever gouty inflammation is transferred to the more important parts, the most timid practitioners are compelled to resort to the fullest activity of depleting treatment. As a general tonic, the Bath waters are again recommended in strong terms. He concludes by a few remarks on the administration of mercury in gout, a practice which he appears to approve; for, though acknowledging that mercury will excite a paroxysm of the disease, and that such paroxysm is usually very violent, he adds—if it be treated according to the principles here laid down, the recovery will be more speedy, and the constitution will be more than commonly relieved by it; and he therefore thinks it may be usefully employed where it is desirable to expedite gouty action.

The next subject treated of is Rheumatism, both acute and chronic, which Dr. Barlow considers as only a modification of the former, occurring in different states of the constitution, as formerly described in the work before us; and this renders it the less necessary to pursue a minute analysis of this portion of the book. In the acute rheumatism, we observe nothing remarkably different from the plan usually pursued by practitioners in general; and the Bath waters are not considered as applicable to this stage of the disease. In the chronic rheumatism, the peculiar state of the constitution is the essential object to study; for in remedying this derangement the cure will consist. In a few words, "To relieve plethora; to excite the constitutional powers to the necessary extent when deficient, or moderate them if in excess; are the great objects to be accomplished. For removing plethora, blood-letting is the direct and speedy remedy; deficient powers are beneficially excited by mercury and the Bath waters; and excessive actions are moderated by evacuations, and the whole antiphlogistic regimen." (p. 124.) In the convalescent state of the patient, allusion is made to shampooing and the course of friction, as originally recommended by Mr. Grosvenor. The doctrines are well illustrated by sixteen cases, but which want of room renders it impossible for us to do more than mention.

A few pages are next devoted to the consideration of Palsy, chiefly for the purpose of advocating the employment of the Bath waters in that disease; but, as a patient who has once experienced an attack is liable to a second, our author enters a little into the remote cause of the disease, which he says is a plethoric state of the system, either absolute or relative, and which can only be remedied by depletion and rigorous diet. In the justness of these sentiments we fully concur, and we willingly add our testimony to his in favour of the Bath waters in these cases.

On Eruptive Diseases Dr. Barlow says but a few words; but what he says is worth repeating. Notwithstanding all the modern arrangements of cutaneous disease, not much, he thinks, has been done to improve our practice; and, as far as regards the great proportion of eruptive diseases, the simple distinction of irritable and indolent seems to be the only practical one worth making. It is equally true, that if the constitution is deranged, it must be put to rights before the eruptions can be cured.

We now draw to a conclusion. A case of Dropsy stands next in order, prefaced by a remark as to the occasional inflammatory origin of this disease,—a point now, we believe, universally acknowledged; so that we are not under the necessity of recommending a practice which is not, we believe, essentially different from that followed by every judicious practitioner.

A few cases of Inflammatory Œdema are subjoined, to show the efficacy of blood-letting and purging in such cases.

An Appendix, occupying fourteen pages, contains a summary of the author's former essay published in the Edinburgh Journal, and which, being so constantly referred to, he considered it would not be unacceptable to his readers for him to transcribe, at least in part.

### DIVISION II.

### FOREIGN.

ART. III.— *Rapport fait à l'Academie des Sciences, par* M. CUVIER, *sur un Mémoire de* M. FLOURENS, *intitulé "Determination des Proprietés du Systeme Nerveux, ou Recherches Physiques sur l'Irritabilité et la Sensibilité."*—(Journal Complémentaire, Mars.)

MM. PORTAL, BERTHOLLET, PINEL, DUMERIL, and CUVIER, were appointed by the Institute to report upon this memoir, which they have considered under three different points of view: viz.—the experiments themselves; the inferences deduced from them; and the language in which they are expressed. M. FLOURENS repeated the principal experiments before the reporters, who were satisfied of their accuracy; and the reasoning appeared in general to be correct; but the language adopted differs in some essential points from that in common use, and might give rise to objections and misunderstandings, if not rectified. The reporters have endeavoured to obviate this difficulty by some criticism and explanations.

When a nerve is pinched or pricked, the muscles on which it is distributed contract with more or less force, and the animal at the same time experiences a greater or less degree of pain. When a nerve is separated from the rest of the system by a ligature or section, and is acted upon in the same manner beneath the ligature or section, the muscular contractions take place as before; but the animal loses the sense of pain, and all power over the action of the muscles supplied by the nerve. A greater attention to the movements which occur in the animal body has shown that it is not by a mechanical action that the nerve influences the muscles: on the contrary, the nerve, during this operation, remains perfectly at rest; and it is not even necessary to employ its intervention, as an irritation applied directly to the muscle itself makes it contract. This effect results for some time in a muscle, the nerve of which is cut; and even if the muscle be removed from the body. It is this property to which GLISSON and HOFFMAN had already attracted the attention of physiologists, which became, about the middle of the eighteenth century, the subject of the experiments of HALLER, and gave rise to the doctrine of *irritability*. These experiments show that this property of contracting with force, whether by immediate irritation or in consequence of irritating a nerve, exists in the muscular fibre, and does not exist in any other animal texture. Their importance excited much interest: the pupils of this great physiologist repeated his experiments, and did not fail to exaggerate the results.

As irritability is not in proportion to the size of the nerves which supply the muscle, and as it was at that time believed that there were muscular parts entirely, or almost entirely, devoid of nerves, some went so far as to think that this property belonged to the muscular fibre itself, independent of the nerves; that these might be agents productive of irritation, but that other irritants could act without them. Haller himself, however, did not embrace this opinion: many very precise passages show that he was by no means ignorant of the co-operation of the nerves in the phenomena of irritability; and the more such phenomena have been studied, the more clearly has this co-operation appeared. At present, when the nerves of every muscular part are known,—when no muscular fibre can be imagined which is not in connexion with a nervous filament,—no one will venture to contend that such nervous filament rests inactive during the contraction. All that is satisfactorily proved is this, that the contraction may take place independent of all sensation in the animal, and of all volition which such sensation might have produced.

Now, this last proposition, which Haller first brought clearly to light, and the natural application he made of it to the involuntary motions, (such as those of the heart and viscera,) overturned completely the physiological system which had been long in vogue,—that of Stahl, which constituted the mind the author of all the movements of the body, not only of those we feel and will, but likewise of those which are insensible to us. The doctrine of Stahl was adopted by Sauvages, and the school of Montpellier was pitted against Haller; but it could only be defended by introducing innovations of language, which for a long time gave to physiology the appearance of being the most difficult, mysterious, and contradictory of all the sciences. The Stahlian doctrine required the idea of sensibility to be generalized to such a degree, as to give this name to every nervous co-operation accompanied with motion, even when the animal had no perception of it. Thus organic and local sensibilities were established, on which reasoning was founded, just as if they had differed in nothing from ordinary and general sensibility. The heart, stomach, womb, &c. according to those physiologists, felt and willed; each organ becoming individually a little animal, gifted with the faculties of the great one. This confusion of terms was singularly favoured and increased by the indefinite meaning which most of these terms have in our language: in fact, "sensible" expresses that which is capable of experiencing sensations,—of exciting them,—or of communicating them. In the first sense, we say that an animal is *sensible;* in the second, we speak of a *sensible* light or sound; and in the third, physiologists call the nerves *sensible.* Writers of much genius have deceived themselves by the use of this figurative language, and of words having a double meaning, so much as to imagine they were explaining phenomena, when they had only changed the ordinary forms of expression into metaphor: and this deception, it is to be supposed, was communicated to many of their readers. Fortunately, however, it did not impose upon men accustomed to habits of strict ratiocination: they gave to each expression a particular meaning, fixed by positive definition, and avoided, with the greatest care, its employment in any other acceptation.

Now, it appears to us that the necessary purposes of science had been sufficiently fulfilled in these times by rigorous physiologists, with regard to the property of which we speak; and that it was unnecessary, on this account, to change the language already adopted. When it is said the muscular fibre is irritable, we understand that it alone can contract on the application of irritants;—when it is said a nerve is not irritable, we understand that irritants do not make it contract; but certainly it is not pretended by that, that it cannot produce irritation in a muscle; —when we say a nerve is sensible, it is understood that the animal receives all sensation through the medium of the nerves; but assuredly it is not meant that the nerve, separated from the body, can continue to give sensations to the animal, and still less that it can of itself experience them.

We would, therefore, advise M. Flourens to strike out of his essay the first part relative to this nomenclature, which cannot but render our ideas more confused, without in any way advancing the interests of science. Thus, because a nerve, on being pricked, excites contractions in a muscle, he concludes that the nerve is *irritable*. It is obvious that in this proposition he teaches nothing new, but merely changes the meaning of the word irritable. Because the nerve, separated from the rest of the system, no longer gives sensation to the animal, he concludes that it is not *sensible*. Here again is simply a verbal change, which expresses nothing but what was known before. M. Flourens is himself aware that he introduces novelties of languages; for he says, "I call *irritability* the property which a nerve has of exciting sensation and motion, without experiencing them itself." Now, it is always dangerous to give a new meaning to a word already understood: it were better to invent a new one, than to distort the old. What is true and independent of verbal disputes is this, that the fibre contracts, whether it be irritated immediately or mediately through the nerve, and consequently that the nerve is a conductor of irritation; that the animal feels the impressions made upon the nerves, when these are in free communication with the encephalon, and consequently that the nerve is a conductor of sensation. Such are the terms of which we shall make use in what follows.

To express, then, in general language, the true questions proposed by M. Flourens, (and which, perhaps, are not sufficiently determined in the title of his memoir,) he endeavours to ascertain by experiment—1. From what points of the nervous system artificial irritation may set off to arrive at the muscle; 2d, to what points of this system an impression must be propagated to produce sensation; 3d, from what points voluntary irritation descends, and what parts of the system must be influenced to produce it regularly.

In the first part, he has only considered the questions in relation to vertebrated animals, and to their nervous system of animal life; that is to say, the brain, the spinal marrow, and the nerves arising therefrom.

The author commences with the nerves, and repeating, with regard to them, the experiments already known, he establishes the two general effects of their irritation, such as we have described above. He shows, in a satisfactory manner, that, in order to effect contraction, a free and continued communication is requisite between the nerve and muscle; and that, to produce sensation, a similar communication with the brain is equally necessary. Hence he concludes, that neither contraction nor sensation belong to the nerve; that these two effects are distinct; that they may take place independent of each other; and that these propositions hold good, at whatever part, and in whatever branch, of a nerve the communication is interrupted. Employing the same method with regard to the spinal marrow, he arrives at similar conclusions. When it is irritated in any given point, contractions are excited in all the muscles which derive their nerves from below this point, if the communication remains free; but not if the communication be intercepted. Exactly the reverse obtains with regard to sensation; and, as in the nerves the government of the will requires the same freedom of communication as sensation, the muscles beneath the intercepted part no longer obey the animal, and he does not feel them: in fine, if the spinal marrow be intercepted at two points, the muscles which receive their nerves from this interval experience contractions alone; but the animal does not command them, nor receive from them any sensation.

We shall not relate all the combinations according to which M. Flourens varied his experiments; it is sufficient to remark, that they all lead to the conclusions we have just mentioned. The author concludes from them, that sensation and contraction belong no more to the spinal marrow than to the nerves; and this conclusion is certain with regard to entire animals.

It would be an important question to determine if it be so generally in respect to animals which have lost their brain, some of which appear far from immediately losing all their animal functions: but this is a question to which we shall have occasion to revert, considered in respect of warm-blooded animals. M. Flourens concludes, in addition, from one part of his experiments, that it is by the communication established between all the nerves by means of the spinal marrow, that what he calls the dispersion, or generalization, of irritations is established,—or, in other words, by which general sympathies are effected: but he has not developed this proposition sufficiently to enable us to appreciate his reasoning upon it.

We now come to the *brain*, and it was from this part of the system that new light was to be expected from experiments better contrived than those of preceding physiologists. Although Haller and his disciples made many trials upon the brain, to discover its vital properties, and the particular functions of the different parts which compose this complicated organ, it may fairly be allowed that these experiments did not lead to satisfactory results; because, on the one hand, neither the connexions of the different parts of the brain, nor the direction and communication of the medullary fibres, were at that time sufficiently known; and because, on the other, these were not sufficiently isolated in the performance of the experiments. When the brain, for example, was subjected to pressure, it could not be known what internal part of it was most powerfully acted upon: when it was punctured, it was not sufficiently ascertained to what depth the instrument penetrated, nor what organ it had entered. M. Flourens has made this objection, with some reason, to the experiments of Haller, Zinn, and Lorry; and he has himself endeavoured to avoid them, in operating principally by the method of ablation ("*ablation*;") that is to say, in removing, wherever it was possible, the part, the precise function of which he wished to determine.

In order to show more clearly the facts which he has ascertained, we shall give a rapid sketch of the parts of which he treats.

It is now ascertained, principally through the late researches of MM. Gall and Spurzheim, that the spinal marrow is a mass of medullary matter, white externally, grey internally, divided longitudinally upwards and downwards by furrows, the two bundles of which communicate together by means of transverse medullary fibres; that it is dilated at intervals; that it gives from each dilatation a pair of nerves; that the *medulla oblongata* is the upper part of the spinal marrow, enclosed in the cranium, which likewise gives rise to various pairs of nerves; that the communicating fibres of its two bundles cross each other in such a manner that those of the right side go to the left, and *vice versa;* that these bundles, after having been first dilated by an admixture of grey matter, and having formed the eminence called *pons varolii,* separate, and take the name of *crura cerebri,* continuing still to give origin to nerves; that they become dilated again by a new addition of grey matter, to form the masses called *optic thalami,* and a third time to form the *corpora striata;* that, from all, the external edge of these last dilatations, arises a plate of greater or less thickness according to the species, completely invested in grey matter, which turns upwards to cover them, forming what are called the *hemispheres,* and which, after being bent down in the middle, unite to that of the opposite side by one or more commissures or bundles of transverse fibres, the most considerable of which, peculiar to the mammalia, is called *corpus callosum.* It is known, besides, that on the crura cerebri behind the optic thalami are one or two pairs of smaller dilatations, known, when there are two pairs, as in the mammalia, by the name of *tubercula quadrigemina,* from the first of which the optic nerves seem to arise; that the olfactory nerve is the only one which does not apparently take its origin either from the medulla or its pillars; finally, that the *cerebellum,* white within and ash-coloured without, like the hemispheres, but often much more divided by external folds, is situated transversely behind the tubercula quadrigemina, and upon the medulla oblongata, to which it is united by transverse bundles, called *crura cerebelli,* which enter at the side of the pons varolii.

It was among these masses, so various and complicated, that it was necessary to seek the point from which irritation departed and the point where sensation arrives; it was their respective co-operation in acts of volition which was to be determined:—such was the chief object of M. Flourens' investigations. He first examined how far up it was possible to go, still producing efficient irritation on the muscular system, and he found a point where these irritations remained ineffectual; then taking the brain at the opposite part, he irritated it at points deeper and deeper, as long as he did not act upon the muscles; and, when he did begin to act upon them, he found himself at the same point where the action had ceased in ascending. This part is also that where the sensation of irritation applied to the nervous system likewise ceases: above this, punctures and wounds do not excite pain. Thus M. Flourens pricked the hemispheres without producing contraction of the muscles, nor the appearance of pain in the animal; he removed them in successive slices: he did the same with regard to the cerebellum; he removed at once the hemispheres and cerebellum. The animal remained passive. The corpora striata and the optic thalami were attacked, and removed without any other effect: the iris was not contracted, nor even paralysed. But, when he pricked the tubercula quadrigemina, trembling and convulsions began, and these increased in proportion as he penetrated into the medulla oblongata. Pricking the tubercles, as well as the optic nerve, produced quick and continued contraction of the iris. These experiments agree with those of LORRY, published in the third volume of the "Memoires des Savans étrangers." "Neither the irritation of the brain nor of the corpus callosum itself produce convulsions: it may even be removed with impunity. The only part among those contained in the brain which has appeared uniformly and universally capable of exciting convulsions, is the medulla oblongata: it is this part which produces them to the exclusion of every other." They contradict the experiments of Haller and Zinn with regard to the cerebellum; but, from what M. Flourens has seen and pointed out, it appears that these physiologists had touched the medulla without being aware of it. The author concludes that the medulla oblongata and the tubercles are (in his language) irritable; which in ours means that they are conductors of irritation, like the spinal marrow and nerves, but that neither the cerebrum nor cerebellum possess this property. The author hence concludes, likewise, that these tubercles form the continuation and superior termination of the spinal cord and medulla oblongata; and this opinion is in conformity with their situation and anatomical connexions.

Wounds of the brain and cerebellum do not excite pain any more than convulsions, and, in ordinary language, we would say that both are insensible: but M. Flourens, on the contrary, says that these are the sensible parts of the nervous system; which means simply that it is to them the impression received by sensible organs must be conveyed, in order that the animal may experience a sensation. M. Flourens appears to have established this proposition in a satisfactory manner, with regard to the senses of sight and hearing. When the cerebral lobe of one side is removed, the animal does not see with the eye of the opposite side, although the iris of this eye retains its mobility: when both lobes are removed, he becomes both blind and deaf. But it does not appear that he has shown it equally well in respect to the other senses. First, he has not, and could not make, any experiment upon smell or taste; and, even with regard to touch, the experiments are unsatisfactory. Indeed, the animal, thus mutilated, assumes the appearance of stupor: he has no longer any will of his own, and performs no voluntary movement; but, when he is struck or pricked, he affects the gait of an animal which awakes: in whatever position he is placed, he resumes his equilibrium. If laid on the back, he rises; if pushed, he walks. A frog leaps when touched; a bird flies if thrown into the air,—it struggles if annoyed,—if water be poured into the beak, it swallows. Without doubt, one can scarcely suppose all these actions to take place without being excited by some sensation. Certainly they are not guided by reason: the animal escapes without object; he has no longer any memory; he strikes himself many times against the same obstacle: but this proves at most (indeed it is the expression of M. Flourens himself,) that such an animal is in a state of sleep. But we are far from thinking that a man who is asleep when he moves, and takes a more convenient position, is altogether deprived of sensation; and, although the perceptions have not been distinct, and although he has not preserved the recollection of them, this is no proof that he has not experienced them. Thus, instead of saying, with the author, that the cerebral lobes are the only organs of sensation, we would restrict ourselves to ascertained facts, and content ourselves with saying that these lobes are the sole receptacle where the senses of sight and hearing can be perfected, and become perceptible to the animal. If we wished to add to this, we would say that they are likewise those where all the sensations take a distinct form, and leave durable traces on the memory,—that they serve, in a word, as the seat of memory; a property, by means of which they furnish the animal with materials for judgment. This conclusion, thus reduced to proper terms, becomes the more probable, in that, besides the verisimilitude which it receives from the structure of these lobes and their connexion with the rest of the system, comparative anatomy offers another confirmation in the constant relation of the volume of these lobes with the degree of intelligence of the animal.

After the effects of ablation, properly so called, M. Flourens examines those of the extirpation of the tubercula quadrigemina. The removal of one of them, after a convulsive movement, which soon ceases, produces, as a permanent result, blindness of the opposite eye and involuntary staggering, (*tourniement;*) that of both tubercles renders the blindness complete, and the staggering more violent and long-continued. The animal, however, retains all its faculties, and the iris continues contractile. The deep extirpation of the tubercle, or the section of the optic nerve only, paralyzes the iris: from which the author infers, that the ablation of the tubercle only acts as the division of the nerve would do; that this tubercle is only a conductor with regard to vision; and that the cerebral lobe alone is the seat of the sensation, the point where it is consummated, and passes into perception. It is necessary to remark, besides, that, in pushing this extirpation of the tubercles very deep, the medulla oblongata becomes concerned, which gives rise to violent and long-continued convulsions.

The part of the author's investigations which possess most characters of interest and novelty, relates to the functions of the cerebellum. During the ablation of the first layers, there appeared only a slight weakness and want of harmony among the movements. At the middle layers, a disturbance nearly general was manifested. The animal, in continuing to see and hear, only executed quick and irregular movements: the faculty of flying, walking, and keeping itself standing, were lost by degrees. When the brain was cut off, this faculty of performing regulated motions had entirely disappeared. Placed upon the back, he did not rise; but continued to see the blow which menaced him; he heard sounds, and endeavoured to shun the danger which was threatened: in a word, feeling and volition were retained, but the power over the muscles was lost; scarcely could he support himself with the assistance of the wings and tail. In depriving the animal of the brain, it was thrown into a state resembling sleep: in removing the cerebellum, it was brought to a state resembling intoxication. "It is surprising," says M. Flourens, "to see the pigeon, in proportion as it loses the cerebellum, gradually lose the power of flying,—then that of walking,—and, lastly, that of standing: this last is only lost by degrees. The animal begins by being no longer able to keep its balance on the legs; then the feet do not suffice to support it, and next every fixed position becomes impossible; it makes incredible efforts to assume some particular position, without effecting it: notwithstanding, when exhausted with fatigue, it appears to wish for repose. Its senses were so much alive, that the slightest gesture made it begin its contortions anew, without the smallest degree of convulsive action being induced, as long as the medulla oblongata and tubercula quadrigemina were left untouched."

(The reporters seem to have supposed these experiments without any precedent in physiological record; but, in a note by the writer in the Journal Complementaire for March, reference is made to a work by M. Rolando, in which similar experiments are described. The inferences, however, drawn by the Italian physiologist, differ in this—that he regarded the cerebellum, not as the regulator, but as the source of locomotion.)

Experiments on the cerebellum of quadrupeds, particularly adults, are extremely difficult, on account of the large portions of bone it is necessary to remove, and the great vessels which require to be cut. The greater number of experimentalists operated after some plan already known, and, for the most part, have been too ready to see what they intended; and assuredly no one has heretofore suspected that the cerebellum was in some manner the regulator of locomotion. This discovery, if repeated experiments, made with necessary precaution, should establish it as a general law, must reflect the highest honour on the young physiologist whose work we have analyzed.

The integrity of the cerebral lobes is necessary to the exercise of sight and hearing: when they are removed, the will no longer manifests itself by voluntary acts. However, when the animal is immediately excited, he performs regular movements, as if endeavouring to avoid pain or inconvenience; but these movements do not effect his purpose, most probably because the memory, which has been removed along with the lobes which constituted its seat, no longer afford grounds or elements of judgment: these movements have no consistency, for the same reason, that the impulse which caused them neither leaves any remembrance nor permanent volition. The integrity of the cerebellum is necessary to the regularity of locomotion: let the brain remain, the animal will see, hear, and have evident and powerful volition; but, if the cerebellum be removed, he will never find the balance necessary to locomotion. As to the rest, irritability remains in parts without the brain or cerebellum being necessary. Every irritation of a nerve brings it into play, in muscles to which it is distributed: every irritation of the spinal marrow excites it in all the members beneath the point of its application. It is quite at the top of the medulla oblongata, at the point where the tubercula quadrigemina joins it, that this faculty of receiving and propagating irritation on the one hand, and pain on the other, ceases. It is this point at which sensation must arrive in order to be perceived: it is from hence that the mandates of the will must emanate. Thus, the continuity of the nervous organ from this point to the parts is requisite for voluntary motion, and for the perception of impressions, whether external or internal.

All these conclusions are not identical with those of the author, and still less are they expressed in the same terms; but they are such as have appeared to the reporters to result from a rigorous examination of the facts he has established.

